# cGAS/STING-mediated upregulation of NKG2D ligands in LSCs contributes to enhanced sensitivity to NK cells

**DOI:** 10.3389/fonc.2026.1764427

**Published:** 2026-02-23

**Authors:** Yan Ouyang, Yilin Qin, Qianmin Zhang, Pengcheng Zhu, Huifang Zhu

**Affiliations:** 1 Neonatal/Pediatric Intensive Care Unit, Children’s Medical Center, First Affiliated Hospital of Gannan Medical University, Ganzhou, China; 2 First Clinical Medical College of Gannan Medical University, Ganzhou, China; 3 Institute of Children’s Medical, First Affiliated Hospital of Gannan Medical University, Ganzhou, China; 4 Ganzhou Key Laboratory of Immunotherapeutic Drugs Developing for Childhood Leukemia, Ganzhou, China

**Keywords:** CGAS, DNA damage response, NK cells, NKG2D ligands, PARP1, STING

## Abstract

**Introduction:**

Natural killer (NK) cells-mediated immune surveillance is essential role for tumor recognition and elimination. The binding of NK group 2 member D (NKG2D) ligands to NKG2D receptor is sufficient to activate the NK cells' cytotoxicity against targeted cells. Here we reported that the inhibition of poly (ADP-ribose) polymerase 1 (PARP1) in leukemia stem cells (LSCs) induced the expression of NKG2D ligands through the activation of DNA damage response.

**Methods:**

Flow cytometry and Quantitative real-time RT-PCR were used to detect the expression levels of NKG2D ligands on cell surface and mRNA levels in leukemia cells, respectively. Cytotoxicity assay was applied to examine the cytotoxic activity of NK cells against leukemia cells. Dual luciferase reporter (DLR) assay was employed to detect the promoter activation of NKG2D ligands. Western blotting was conducted to check the protein expression level.

**Results:**

Activated ataxia-telangiectasia mutated proteins (ATM) and ɣH2AX foci accumulation were observed under the treatment of PARP inhibitor, leading to the accumulation of damaged DNA. Subsequently, cyclic GMP-AMP synthase (cGAS) was activated, and stimulator of interferon gene (STING) was recruited to promote the downstream signal transduction via TANK-binding kinase-1 (TBK1) and transcription regulatory factor 3 (IRF3). However, the NKG2D ligands induced by PARP inhibitor was reduced in STING or IRF3-knockdown LSCs, indicating that STING and IRF3 were necessary for the regulation of NGK2D ligands in LSCs upon the DNA damage response.

**Discussion:**

These data indicated that the DNA damage response-mediated cGAS/STING/TBK1/IRF3 signalling pathway played an essential role in modulating NKG2D ligands expression in LSCs.

## 1 Introduction

Acute myeloid leukemia (AML) is a severe hematological malignancy. According to the cancer statistics of 2025, AML is estimated to be the first leading cause of death and account for the second morbidity among leukemia in the United States (1), making it to be a major global public health concern and bringing heavy burden to the society. AML is characterized by clonal growth and the accumulation of functionally immature blasts, which named leukemic stem cells (LSCs) and first identified in human leukemia cells by Jim Griffin and colleagues (2, 3). LSCs are a subpopulation of leukemia cells that exhibit the stem cell properties with capacity for self-renewal, and the immunophenotype is similar to hematopoietic stem cells (CD34^+^, CD38^-^, CD71^-^, and HLA^-^DR^-^) (4). Recently, increasing evidence suggests that the existence of LSCs is responsible for the drug resistant and leads to the occurrence of Minimal Residual Disease (MRD) after chemotherapy, thus facilitating cancer recurrence (5). Therefore, targeted treatment of LSCs is considered to be a promising strategy to reduce the recurrence rate and improve both survival and cure rates for AML patients.

Natural killer (NK) cells are essential innate immune effectors and accountable for the immunosurveillance of tumorigenesis. They are large granular lymphocytes that responsible for tumor recognition and eradication through a direct killing effect without the need for antigen presentation. The hyperactivation of NK cells are found to inhibit the development of AML *in vivo* (6), and NK cell-based immunotherapy is demonstrated to be a safe and effective therapy for AML patients (7, 8). The cytotoxic function of NK cells is strictly controlled and balanced through the activating and inhibitory receptors, among which, the Natural Killer Group 2D (NKG2D) receptor is one central activating receptor to activate NK cells by recognizing a wide range of ligands, including MHC class I chain-related proteins A and B (MICA and MICB) and members of the ULBP family (ULBP1-6) in humans, and retinoic acid early transcript (Rae-1), UL16-binding protein-like transcript 1 (Mult1), and H60 in mice (9). These ligands are stress-inducible expressed in transformed cells, which arouse the cytotoxicity of NK cells. Therefore, the NKG2D-NKG2D ligands axis plays a crucial role for NK cells to kill cancer cells and is supposed to be a promising target therapeutic strategy in NK cell-based immunotherapy. However, the stress response pathway is usually dysregulated in tumor progression, leading to downregulation of NKG2D ligands on tumor cell surface, which contributes to the tumor immune evasion towards NK cells. AML cells can attenuate their NKG2D ligands expression to undermine NK cells-mediated immunosurveillance, and the reduced NKG2D ligands is associated with poor prognosis in AML patients (10); especially, the absence of NKG2D ligands is supposed to be an essential characteristic for LSC (11). Hence, the understanding of the regulatory mechanisms involved in NKG2D ligands expression in LSCs will help to shed light on the application of NK cell-based immune therapy against AML.

The DNA damage response (DDR) is a highly coordinated network to maintain genomic integrity by initiating DNA repair or inducing cell death in severe conditions. It plays essential roles for cell survival and proliferation. In recent years, the DDR pathway is also demonstrated to be adopted by various tumor cells to regulate NKG2D ligands expression, such as Hodgkin lymphoma and breast cancer (12, 13). Evidence from Stephan Gasser *etal.* suggested that the treatment of DNA-damaging agents or DNA synthesis inhibitors in non-transformed cells, which would initiate the DNA damage response was capable to induce NKG2D ligands expression through the protein kinases ataxia telangiectasia and Rad3 related (ATR) and ataxia telangiectasia mutated homolog (ATM)-dependent manner (14); afterwards, they found that the induction of Rae1 and Mult1 in mouse lymphoma cells was dependent on STING (stimulator of interferon (IFN) genes)-mediated cytosolic DNA sensor pathway, and the transfection of DNA enabled RAE1 expression in NGK2D ligand-negative cells (15). Moreover, the capacity of chemotherapy or radiotherapy to promote NKG2D ligands expression in types of cancers was also demonstrated to be dependent on the induction of DNA damage in tumor cells (16–18). It is strongly recommended that the activation of DDR pathway, as well as the downstream DNA sensors-mediated innate immune signaling pathway is critical for the induction of NKG2D ligands in cells under stress.

In our study, we demonstrated that the inhibition of poly (ADP-ribose) polymerase 1 (PARP1) by inhibitor (PARPi) or RNA interference (RNAi) in LSCs could induce NKG2D ligands expression on cell surface, which subsequently sensitized NK cells’ cytotoxicity against LSCs. PARP1 is one of the most important ADP-ribosyl transferase that is widely implicated in DNA damage and DNA repair events (19). We found that the inhibition of PARP1 by special inhibitor (PARPi) or shRNA leads to the activation of ATM-mediated DNA damage response, which subsequently upregulated the expression cyclic GMP-AMP synthase (cGAS) and promoted the activation of STING. Afterwards, activated STING recruited the protein kinase TANK-binding kinase 1 (TBK1) to promote the phosphorylation and activation of transcription factor interferon regulatory factor 3 (IRF3), leading to the induction of NKG2D ligands in LSCs. The knockdown of STING or IRF3 significantly reversed the effects of PAPRi on the upregulation of NKG2D ligands in LSCs. Taken together, these findings revealed that the cytosolic cGAS-STING signaling pathway was essential for LSCs to regulate NKG2D ligands expression on the cell surface. Our study not only uncovers the underlying regulatory mechanisms involved in the expression of NKG2D ligands in LSCs, but also provides novel insights into the development of NK cell-based immunotherapy against AML.

## 2 Materials and methods

### 2.1 Cell lines and cell culture

Human NK92 MI cells and human acute myeloid leukemia (AML) cell line HL60 were obtained from Shanghai Institute of Biochemistry and Cell Biology, Chinese Academy of Sciences (Shanghai, China); human AML cell lines KG-1α and Kasumi-1 were purchased from ATCC (American Type Culture Collection) (Manassas, VA). NK92 MI were cultured in α-MEM (Alpha Minimum Essential Medium), complemented with 12.5% fetal bovine serum (FBS) (Gibco, Australia) and 12.5% horse serum (Gibco, Australia). HL60, Kasumi-1 and KG1α were cultured in IMDM (Iscove’s Modified Dulbecco’s Medium) complemented with 15% FBS (Gibco, Australia).

### 2.2 Isolation of primary human AML cells and NK cells

Primary AML cells were obtained from the bone marrow (BM) samples in clinical AML inpatients in the department of hemopathy in the First Affiliated Hospital of Gannan Medical University. The research protocol had been reviewed and approved by the Research Ethics Committee of the First Affiliated Hospital of Gannan Medical University, with Ethics No. LLSC-2023-353. Briefly, mononuclear cells were separated from BM by density-gradient centrifugation with Ficoll-Hypaque (Cytiva, Uppsala, Sweden), and cultured in IMDM (HyClone, Logan, Utah, USA) supplemented with 15% FBS (Gibco, Australia), 4 mM L-Glutamine (Gibco, Brazil), 100 ng/mL human IL-3 (Proteintech, China), 10 ng/mL human IL-6 (Proteintech, China), 50 ng/mL human M-CSF (Proteintech, China), TPO (Proteintech, China), 50 ng/mL human FLT3-ligand (Proteintech, China), 50 ng/mL human SCF (Proteintech, China) and 1% penicillin and streptomycin.

Human peripheral blood mononuclear cells (PBMCs) were firstly separated by density-gradient centrifugation with Ficoll-Hypaque (Cytiva, Uppsala, Sweden) from healthy donors, and then primary NK cells were isolated from PBMC by human NK Cell Isolation Kit (Cat#: 130-092-657, Miltenyi Biotec) via untouched negative MACS Separation as instructions. The purity of NK cells was analyzed by flow cytometry (see details in the supplementary information). Primary NK cells were cultured in RPMI-1640 Medium, complemented with 10% FBS (Gibco, Australia), 200 IU/mL rhIL-2 (Beyotime, China), 10 ng/mL rhIL-15 (Beyotime, China) and 1% penicillin and streptomycin.

### 2.3 Isolation of leukemia stem cells

The LSCs were isolated from KG-1α cells and primary AML cells by CD34^+^CD38^–^ Cell Isolation Kit (Cat#: 130-114-822, Miltenyi Biotec) via MACS Separation as instructions. Briefly, KG-1α cells or primary AML cells were collected in cold medium by centrifuge at 300×g for 10 minutes, following by the steps sequentially: First magnetic labeling (incubation with CD34 MultiSort MicroBeads)-First magnetic separation-Removal of MultiSort MicroBeads and second magnetic labeling (incubation with CD34 MultiSort MicroBeads and CD38 MicroBeads)-Second magnetic separation with LS Columns. After these steps, the target LSCs expressed CD34^+^CD38^–^ were isolated, and analyzed by flow cytometry to identify the expression level of CD34 and CD38 (see details in the supplementary information).

### 2.4 Reagents and antibodies

Inhibitors for PARP1/2 (Talazoparib Tosylate, Cat#: HY-108413) and ATM (CGK733, Cat#: HY-15520) were purchased from MCE. Antibodies against PARP1 (Cat #: 66520-1-Ig, Proteintech), cGAS (Cat**#**: 79978, Cell signaling technology), STING (Ca**#**: 13647, Cell signaling technology), phospho-STING (Ser366) (Cat#:72650, Cell signaling technology), phospho-TBK1 (Ser172) (Cat#: 5483, Cell signaling technology), TBK1 (Cat#: 3504, Cell signaling technology), IRF3(Cat#: 4302, Cell signaling technology), phospho-IRF3 (Ser396) (Cat#: 4947, Cell signaling technology), ATM (Cat#: 27156-1-AP, Proteintech), phospho-ATM (Ser1981) (Cat#: 5883, Cell signaling technology), phospho-γH2AX (Ser139) (Cat#: 83307-2-RR, Proteintech), H2AX (Cat#: 39689, Proteintech), GAPDH (Cat#: 60004-1-Ig, Proteintech), α-Tubulin (Cat#: 14555-1-AP, Proteintech) were used as primary antibodies in the western blotting assay; Goat Anti-Mouse IgG antibody, HRP conjugated (Cat#: 2900264, Millipore), and Goat Anti-Rabbit IgG antibody, HRP conjugated (Cat#: 3256751, Millipore) were used as second antibodies. Primary antibodies against human NKG2D ligands (MICA/B, ULBP1, ULBP2/5/6, and ULBP3) using in flow cytometry were purchased from R&D System, with Cat. No. as follows: FAB13001A, FAB1380A, FAB1298A, and FAB1517A, respectively; mouse anti-NKG2D antibody (Cat#: MAB139) used in functional blockade for NKG2D receptor and isotype control Goat anti-mouse IgG2A-APC (Cat#: IC003A) were obtained from R&D System. Mouse IgG (Cat#: SA00001-1) was purchased from Proteintech.

### 2.5 Western blotting

LSCs treated with PARPi were lysed on ice with RIPA buffer supplemented with Protease Inhibitors, phosphatase inhibitors (Cat#: 4693116001, Roche) and Phenylmethanesulfonyl fluoride (Cat#: HY-B0496, MEK). After a quantification for protein concentration using BCA assay kit (Cat#: 23227, Thermo Fisher), the lysates were subjected to the SDS-PAGE electrophoresis. The WB analysis was performed as previously described (20). The blots were observed using a Chemiluminescence Imaging System (SCG-W3000, Servicebio)

### 2.6 Plasmids construct

Restriction endonuclease and T4 DNA ligase were obtained from New England Biolabs (Ipswich, MA). The pLKO.1 vector was used to construct vectors expressed shRNAs targeting human PARP1, IRF3 and STING, with sequences synthesized by Genecreate (Wuhan, China) as follows: PARP1-1#: CTTCGTTAGAATGTCTGCCTT, PARP1-2#: CTCTCAAATCGCTTTTACA; STING-1#: GTCCAGGACTTGACATCTTAA, STING-2#: CATGGTCATATTACATCGGAT; IRF3-1#: GATCTGATTACCTTCACGGAA, IRF3-2#: GCAGGAGGATTTCGGAATCTT. The shRNA-carrying lentiviruses were produced in HEK293T cells by co-transfecting with packaging plasmids pCMV-dR8.91 and pMD2.G. PARP1, STING or IRF3 stable knockdown LSCs were established by lentiviruses infection, puromycin screening, and limited dilution.

Promoter fragments of human ULBP1 (601 bp), ULBP3 (501 bp), MICA (647 bp) and MICB (611 bp) were amplified by PCR from human genomic DNA using the following primers: ULBP1 forward, 5’-TGC*GGTACC*CCATTCACTCCCACAACTG-3’, ULBP1 reverse, CTG*AAGCTT*AGGAGGGGCCTGTGTGCAC; ULBP3: forward, 5’-CGC*GGTACC*AAAACATGCCTGAGTCTTG-3’, ULBP3 reverse, 5’-CGT*AGATCT*CCCCAAGCAGGACAGGAG-3’; MICA forward, 5’-TGC*GGTACC*ACTCACCCGGATCCGAATC-3’, MICA reverse, 5’-CTA*AAGCTT*GGCCCAGCGCGGTCGCTTAC-3’; MICB forward, 5’-TGC*GGTACC*TCAGATTCCAGATCGCGCTC-3’, MICB reverse, 5’-CTG*AAGCTT*GCGCGGTCGCTCACAAAAT-3’. The PCR products were subcloned into the *KpnI* and *HindIII* or *BglII* sites of pGL3-Basic vector (Promega). The integrity of constructs was confirmed via DNA sequencing by Genecreate (Wuhan, China).

### 2.7 Transfection and dual luciferase reporter assays

The ULBP1-, ULBP3-, MICA-, MICB-promoter reporter plasmid or pGL3-Basic plasmid and the internal control plasmid pRL-TK (Promega), along with pCMV-flag-IRF3/5D plasmid (a gift plasmid) (21) or pCMV-flag empty vector were transfected into HEK293T cells using Lipofectamine 3000 (Invitrogen) according to the manufacturer’s instructions. After 24 hours, luciferase assays were performed with a dual-specific luciferase assay kit (Cat #: E1910, Promega) as described in our previous studies (22).

### 2.8 Flow cytometry analysis

The protein level of NKG2D ligands expressed on cell membrane surface was detected by flow cytometry using FACS Calibur flow cytometer (BD Biosciences) as previously described (23). Data was analyzed by FlowJo_v10.9.0 analysis software.

### 2.9 Quantitative real-time RT-PCR

Total RNA was extracted from LSCs using the Total RNA Extraction Reagent (Cat#: BSC51M1, BioFlux). The samples were subjected to reverse transcription using First Strand cDNA Synthesis Kit (Roche). SYBR Master Mix (Cat#: BSB25L1B, BioFlux) was used in a two-step PCR program (94 °C for 15 s and 60 °C for 60 s) for quantitative PCR. GAPDH was applied as internal standardizer to normalize the gene transcriptional level. Primers targeted human NKG2D ligands were listed as previously described (20).

### 2.10 Cytotoxicity assay

The cytotoxic activity of NK92 MI or primary NK cells was examined by Cytotoxicity Assay Kit as previously described (20). Briefly, NK92 MI cells pretreated with NKG2D neutralizing antibody or control IgG were co-incubated with target cells at the indicated effector (E, NK92 MI cells)/target (T, AML or LSCs) ratio in CO_2_ incubator for 4 hours. Then lactate dehydrogenase (LDH) released from the targeted cells killed by NK92 MI were detected followed the kit instruction (Cat#: G1780, Promega).

### 2.11 Immunofluorescence staining

After the treatment of PARPi, LSCs were placed on glass slides and fixed with 4% paraformaldehyde for 20 min. Cells were permeabilized with 0.1% Triton in PBS for 30 min at room temperature, and incubated overnight in blocking solution containing antibody against γH2AX (Cat#: 83307-2-RR, Proteintech). Fluorescein (FITC)-labeled Goat Anti-Mouse IgG (Cat#: SA00003-2, Proteintech) was employed as a secondary antibody for observation. The nucleus was dyed with DAPI, and evaluated via a Zeiss fluorescence microscope. Images were processed via Adobe Photoshop.

### 2.12 Clinical data set analysis

Microarray data GSE127200 came from the Gene Expression Omnibus (GEO) was chosen to analyze correlation between PARP1 and NKG2D ligands. The effects of NKG2D ligands on the survival of AML patients were analyzed through the online survival analysis database Kaplan Meier plot from TCGA (http://kmplot.com/analysis/index.php?p=background); the effect of PARP1 on AML patients survival was conducted in UALCAN (http://ualcan.path.uab.edu/index.html) according to the instruction.

### 2.13 Statistical analysis

Data was shown as mean ± standard deviation. Two-tailed Student’s t-test was performed for statistical analysis between two groups; one-way ANOVA analysis was used for statistical analysis among groups more than three.

## 3 Results

### 3.1 NKG2D ligands expression level is correlated with the prognoses of AML patients

**Figure 1:**
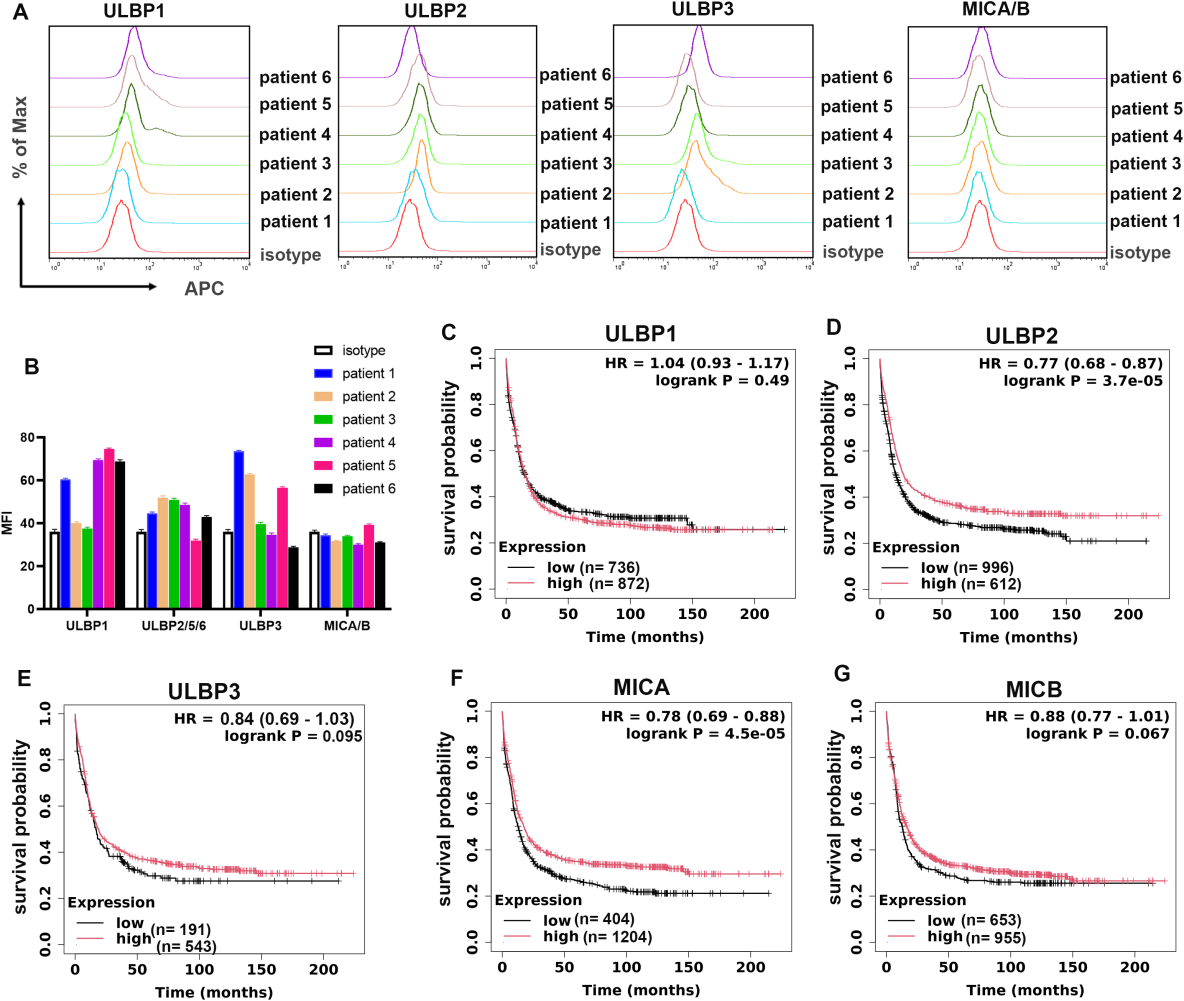
High expression of NKG2D ligands contributes to longer survival in AML patients. **(A)** The expression level of ULBP1, ULBP2, ULBP3 and MICA/B on primary AML cell surface from patients was detected by flow cytometry (BD FACSCalibur). Mouse IgG-APC was used as isotype control. **(B)** The MFI (Median Fluorescence Intensity) value was quantified from **(A)**. **(C–G)** Kaplan-Meier analysis of overall survival of AML patients stratified by the expression levels of PAPR1. Numbers of patients, log-rank p-value and Hazard Ratio (HR) value were shown.

NKG2D ligands are widely expressed on tumor cell surface in solid and hematologic malignancies, which are closely related to its immunogenicity responding to NK cells. However, during the progression of cancers, the expression level of NKG2D ligands is downregulated or absent on cell surface, leading to an immune evasion from the surveillance of NK cells. AML is one of the most severe hematologic tumors with high incidence to be relapsed. It is predicated that the absence of NKG2D ligands may be an immune escape strategy for AML to break the immune balance to promote the development and progression of tumor. To find the expression level of various subtypes of NKG2D ligands on AML patients, primary AML cells were isolated from the BM and checked by flow cytometry. As data shown in **Figures 1A, B**, MICA and MICB were nearly undetectable on AML cell surface from all patients, with ULBP1, ULBP2 and ULBP3 also low expressed. To further evaluate the correlation between NKG2D ligands expression level and AML patients’ prognosis, the survival analysis plotters from AML patients with low and high NKG2D ligands were drawn based on the dada in public online database (http://kmplot.com). It was shown that although the survival time was not significantly different between ULBP1 low expression and high expression AML patients (*P* = 0.49) (**Figure 1C**), patients with high level of ULBP2, ULBP2, MICA and MICB gained a longer survival time than the low-expressed patients (**Figures 1D–G**), indicating that NKG2D ligands could be good predictors for prognosis in AML patients.

### 3.2 LSCs is out of response to NK cells

NK cells-mediated immunotherapy is a promising treatment against leukemia. The relapse of AML is traced back to the presence of LSCs in the bone marrow tumor microenvironment (24). LSCs is a unique population of therapy-resistant leukemia cells with long-term self-renewal capacity (25). Therefore, the targeted therapy against LSCs is considered to be an effective treatment to cure leukemia.

**Figure 2:**
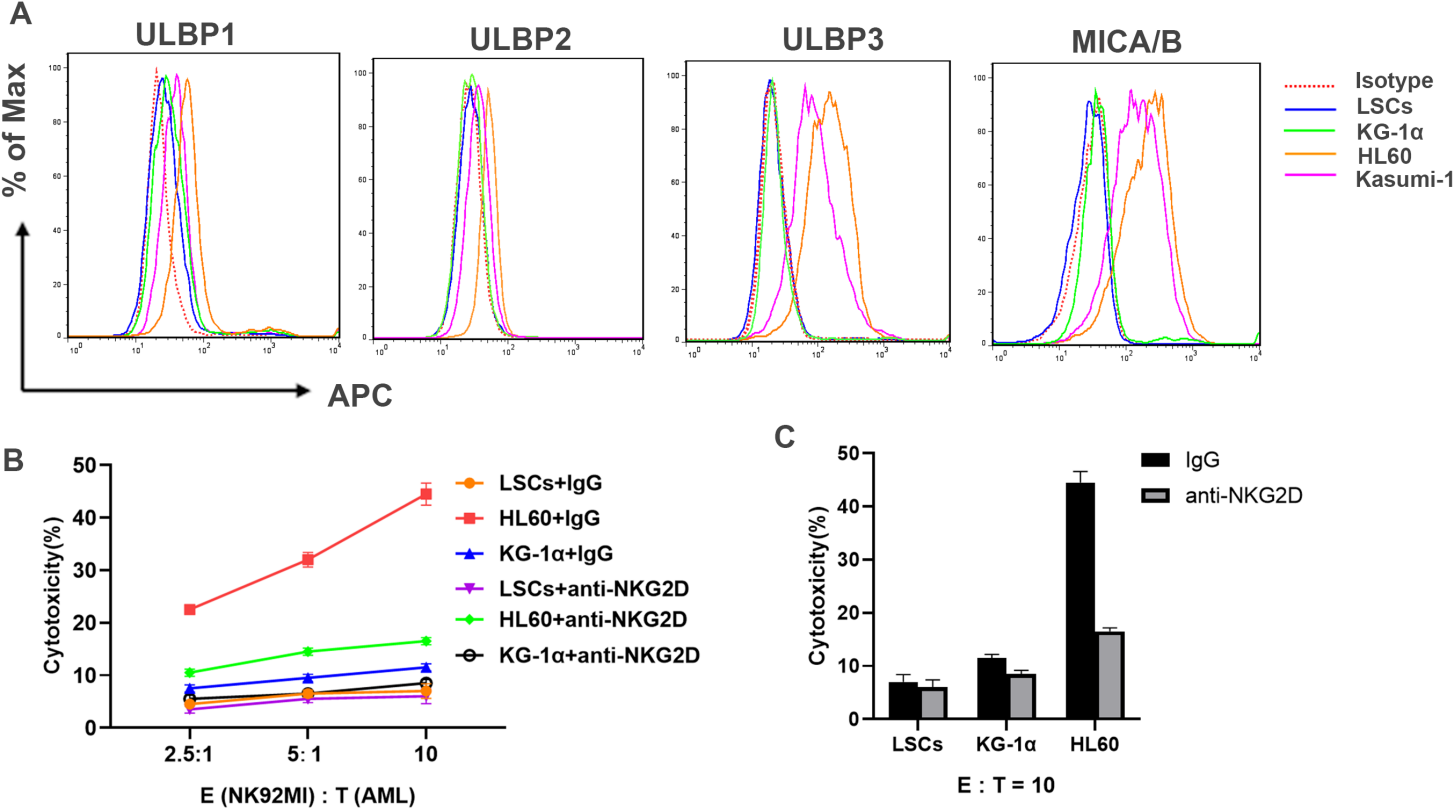
LSCs is out of response to the cytotoxicity of NK cells. **(A)** The expression level of ULBP1, ULBP2, ULBP3 and MICA/B on the cell surface of KG-1α, Kasumi-1, HL60, and LSCs (enriched from KG-1α) were detected by flow cytometry (BD FACSCalibur). Mouse IgG-APC was used as isotype control. **(B)** LSCs, KG-1α and HL60 were co-cultured with NK92 MI cells pretreated with IgG or anti-NKG2D antibody at E:T ratio of 2.5:1, 5:1, and 10:1, respectively for 4 h The cytotoxic activity of NK92 MI cells were measured by Non-Radioactive Cytotoxicity kit according to the released LDH from target cells. Results were representative of three different experiments. **(C)** Quantification of the cytotoxicity of NK92 cells from **(B)** at E:T ratio of 10:1.

It was reported that the NKG2D ligands are absent on the cell surface of LSCs (26). KG-1α is one type of stem cell-like, which is reported to be resistant to most chemotherapeutic drugs (27). We isolated LSCs from KG-1α by magnetic beads to obtain the CD34 positive expression and CD38 negative expression cell population (**Supplementary Figure S1**). Thereafter, we checked the expression level of subtypes ligands by flow cytometry. It was found that, all five subtypes of ligands including ULBL1~3, and MICA/B were not detected on the surface of LSCs, while the NKG2D ligands were expressed on AML cell lines including HL60 and Kasumi-1 (**Figure 2A**). Furthermore, we examined whether LSCs could arouse the cytotoxic activation of NK92 MI through the NKG2D receptor-ligands axis. As shown in **Figures 2B, C**, compared with HL60 cells, NK92 MI cells exhibited poorer cytotoxicity against KG-1α; especially when co-incubated with LSCs, NK92 MI cells nearly exhibited no cytotoxicity, indicating that LSCs lost their ability to sensitize NK cells’ activity. Altogether, these data demonstrated that the absence of NKG2D ligands assisted LSCs to escape from NK cells’ immune killing.

### 3.3 PARP1 is negatively correlated with NKG2D ligand expression in LSCs

**Figure 3:**
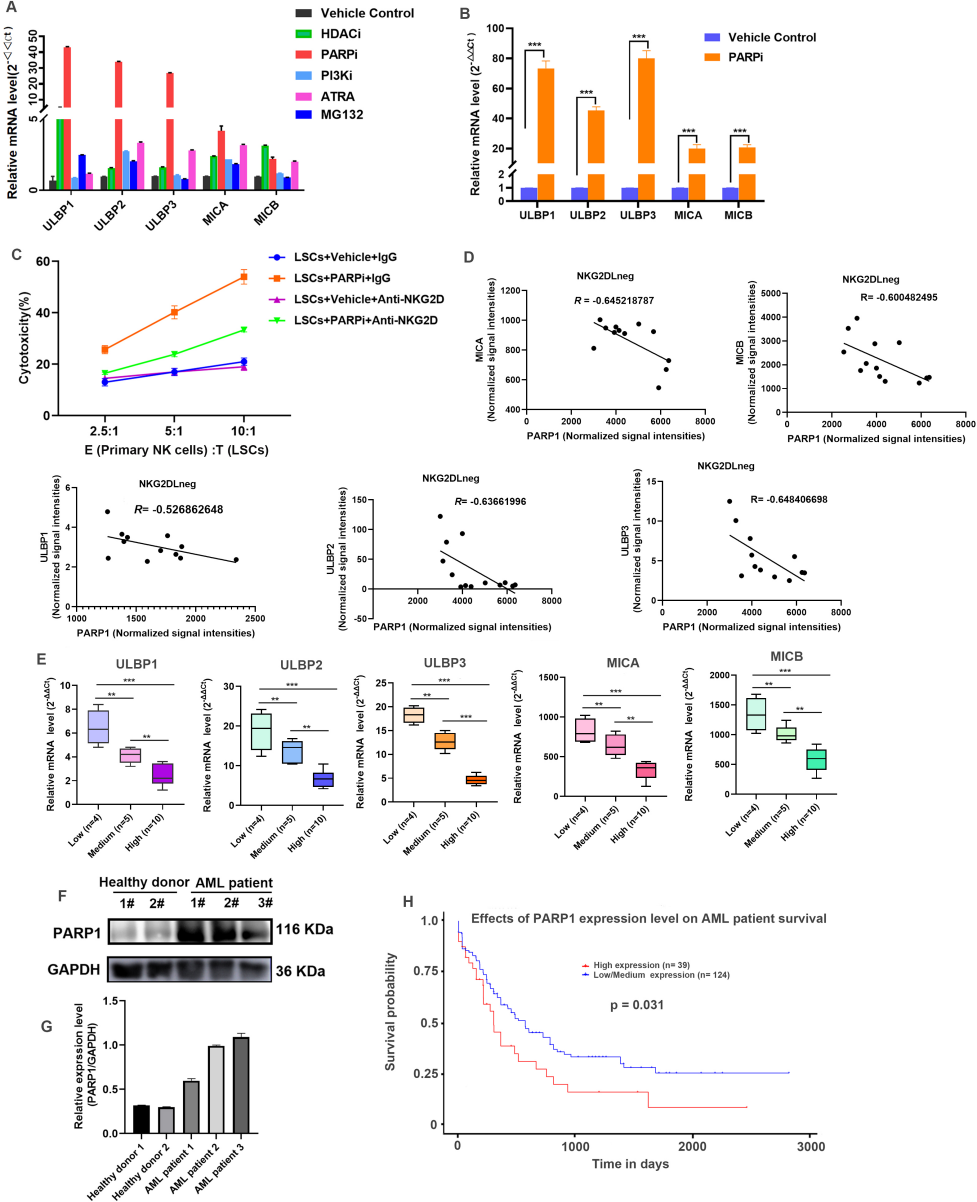
PARP1 is negatively correlated with NKG2D ligands expression in AML patients. **(A)** LSCs were treated with the indicated compounds for 24 hours, and then cells were collected to extract total RNA. The mRNA transcriptional level of ULBP1, ULBP2, ULBP3, MICA and MICB were detected by quantitative real-time RT-PCR. Results were representative of three different experiments. **(B)** Primary LSCs isolated from patient 3, 5, 8 and 11 were treated the PARPi at a concentration of 1 μM for 24 hours, and then the quantitative real-time RT-PCR was performed as **(A)** to measure the transcriptional level of ULBP1, ULBP2, ULBP3, MICA and MICB. Results were representative of three different experiments. **(C)** LSCs were treated with PARPi at a concentration of 1 μM for 24 hours, and then co-cultured with primary NK cells pretreated with IgG or anti-NKG2D antibody at E:T ratio of 2.5:1, 5:1, and 10:1, respectively for 4 hours. The cytotoxic activity of primary NK cells was measured by Non-Radioactive Cytotoxicity kit according to the released LDH from target cells. Results were representative of three different experiments. **(D)** The correlation between PARP1 and NKG2D ligands (ULBP1, ULBP2, ULBP3, MICA and MICB) in NKG2D ligands-negative (NKG2DLneg) AML patients from microarray data set GSE127200 were analyzed by R cor.test. The Pearson Correlation Coefficient also known as *R* value was shown. **(E)** The mRNA transcriptional level of ULBP1, ULBP2, ULBP3, MICA and MICB in AML patients stratified by the expression levels of PAPR1 (Low (n=4), Medium (n=5), and High (n=10)) were detected by quantitative real-time RT-PCR. Data was shown as box plot. The statistical significance was determined by one-way ANOVA analysis (* p < 0.05, ** 0.001 < p < 0.01, *** p < 0.001). All experiments were repeated three times. **(F)** The expression level of PARP1 in PBMCs from healthy donors (n=2) and primary AML cells from AML patients (n=3) were detected by WB. GAPDH was employed as an internal loading control. **(G)** Quantification of the WB shown in **(E)**. The Integrated Optical Density of bands was measured by the software ImageJ. Data was presented as mean ± SD from three experiments. **(H)** Kaplan-Meier analysis of overall survival of AML patients stratified by the expression levels of PARP1. Numbers of patients and log-rank p-value are shown.

Multiple signaling pathways are implicated in the modulation of NKG2D ligands expression in various cancers. However, the regulatory mechanisms were not clearly clarified in LSCs. Some cancer treatments especially chemotherapy and targeted therapies were widely reported to induce NKG2D ligands expression in tumor cells. To explore the potential regulatory strategy in LCSs, a broad screening of small molecules that promoted NKG2D ligands expression in LSCs derived from KG-1α was performed by quantitative real-time PCR. Intriguingly, PARP1/2 inhibitor Talazoparib Tosylate was demonstrated to significantly induce the transcription of ULBP1, ULBP2 and ULBP3 (parts of results from the screening were shown in **Figure 3A**). To further confirm the effects of PARPi on NKG2D ligands, two other PARP1/2 inhibitors Olaparib and Niraparib were also employed in LSCs. It was shown that all these PARP inhibitors obviously promoted the transcription of NKG2D ligands in LSCs (**Supplementary Figure S2**). In primary LSCs (**Supplementary Figure S3**), the transcriptional levels of NKG2D ligands were also found to be upregulated (**Figure 3B**).

Meanwhile, the cytotoxic activity of primary NK cells from healthy donor (**Supplementary Figure S4**) was examined when co-incubated with LSCs pretreated with PARPi (Talazoparib Tosylate). As data shown in **Figure 3C**, under the treatment of PAPRi, LSCs significantly enhanced the cytotoxicity of primary NK cells; however, the blockade of NKG2D receptor by primary antibody against NKG2D in primary NK cells obviously reversed its cytotoxicity towards LSCs even with the treatment of PARPi, indicating that the improved cytotoxic activity for primary NK cells against LSCs was dependent on the increasing expression of NKG2D ligands in LSCs, as well as the binding of ligands to NKG2D receptor.

To understand the role of PARP1 on the regulation of NKG2D ligands in AML, we chose a microarray dataset GSE127200 to analyze the correlation between PARP1 and NKG2D ligands from AML patients. As data shown in **Figure 3D**, the transcription of ULBP1, ULBP2, ULBP3, MICA and MICB were negatively correlated with PRAP1 in NKG2D ligands-negative cells. We further measured the transcriptional mRNA level of PARP1 and NKG2D ligands in AML cells from our hospitalized patients using qPCR assay, and three groups: low, medium, and high were divided according to the transcriptional level of PARP1 (**Supplementary Table S1**). As data shown in **Figure 3E**, the transcription of ULBP1, ULBP2, ULBP3, MICA and MICB was decreased with the increasing of PARP1, suggesting that PARP1 was also negatively correlated with NKG2D ligands in our AML samples.

We next checked the protein expression level of PARP1 in AML patients. As data shown in **Figures 3F, G**, compared with PBMCs from the healthy donors, PARP1 was significantly increased in primary AML cells derived from BM in AML patients (**Supplementary Figure S5**). Also, AML patients with PARP1 low expression had a longer survival compared with those PARP1 high expression patients (**Figure 3H**), indicating that PARP1 was a poor prognostic factor for AML patients. Taken together, these data indicated that PARP1 displayed a negative correlation with NKG2D ligands in AML patients. The inhibition of PAPR1 in LSCs would increase NK cell-mediated cytotoxicity towards LSCs.

### 3.4 The DNA damage pathway is implicated in the regulation of NKG2D ligands expression in LSCs

**Figure 4:**
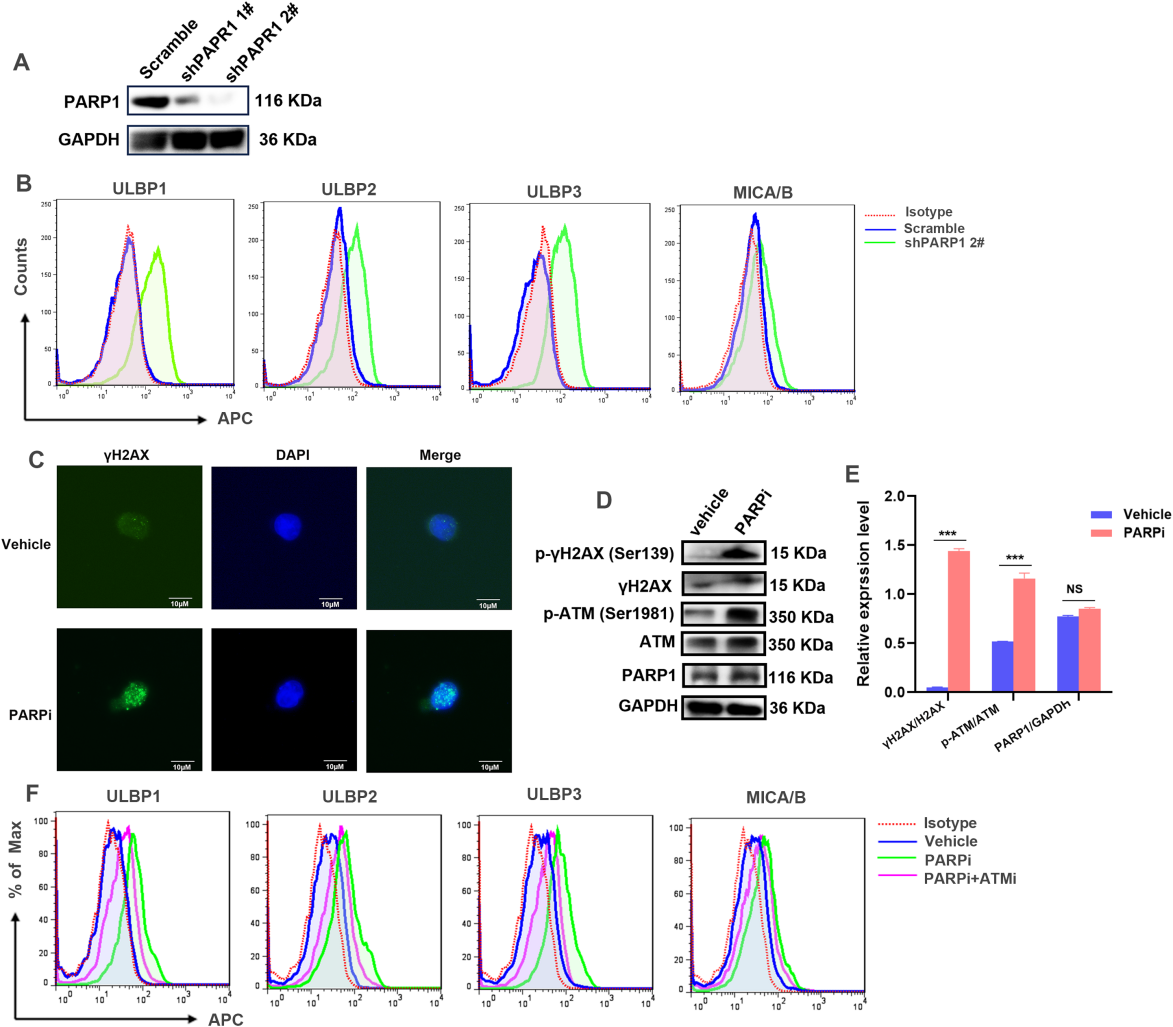
PARP1 is involved in the regulation of NKG2D ligand expression in LSCs. **(A)** WB was used to detect the knockdown efficiency of PRAP1 in LSCs. GAPDH was employed as an internal loading control. **(B)** The expression level of ULBP1, ULBP2, ULBP3 and MICA/B in PARP1-stable knockdown or -scramble control LSCs were detected by flow cytometry (BD FACSCalibur). Mouse IgG-APC was used as isotype control. **(C)** Formation of γH2AX foci mediated by the pretreatment of 1 μM PARP1 inhibitor (PARPi) for 6 hours in LSCs was detected by immunofluorescence and observed in a fluorescence microscope (Zeiss). DMSO was used as vehicle control. Scale bars, 10 μm. **(D)** The protein expression level of γH2AX, H2AX, phosphorylated-ATM (Ser1981), ATM, and PARP1 in LSCs pretreated with 1μM PARPi or Vehicle for 6 hours were detected by WB. GAPDH was employed as an internal loading control. **(E)** Quantification of the WB shown in **(D)**. The Integrated Optical Density of bands was measured by the software ImageJ. Data was presented as mean ± SD from three experiments. **(F)** The expression level of ULBP1, ULBP2, ULBP3 and MICA/B on the cell surface of LSCs treated with 1 μM PARPi or co-treated with 1 μM PARPi and 1 μM ATM inhibitor (ATMi) for 6 hours were measured by flow cytometry (BD FACSCalibur). DMSO was used as Vehicle control. Mouse IgG-APC was used as isotype control. ***p < 0.001. ns, no significance.

We further detected the regulatory roles of PARP1 on NKG2D ligands in LCSs. A PARP1stable knock-down cell line was established in LSCs derived from KG-1α cells, and the knock-down efficiency was checked by WB (**Figure 4A**; **Supplementary Figure S6**). The knock-down of PARP1 could obviously increase the expression level of ULBP1, ULBP2, and ULBP3 in LSCs (**Figure 4B**).

PARP1 is a pivotal regulator in the DNA repair process. The inhibition of PARP1 will result in the accumulation of damaged DNA, which further activates the DDR pathways (28). We next detected whether the induction of NKG2D ligands in LSCs was associated with the activation of DDR pathway caused by PAPR1 inhibition. The immunofluorescence images showed that the inhibition of PARP1 increased γH2AX foci accumulation (**Figure 4C**). In addition, WB analysis revealed that the inhibition of PARP1 elevated γH2AX expression, as well as the phosphorylated forms ATM (**Figures 4D, E**; **Supplementary Figure S7**). However, compared with the inhibition of PARP1 itself, the combined inhibition of ATM (CGK733) impeded the upregulation of NKG2D ligands expression induced by PARP1 inhibitor (**Figure 4F**). Hence, these data suggested that the knock-down or inhibition of PARP1 activated the intracellular DDR pathway, which further increased the expression of NKG2D ligands in LSCs.

### 3.5 The upregulation of NKG2D ligands is associated with the activation of cGAS/STING/IRF3 pathway in AML

**Figure 5:**
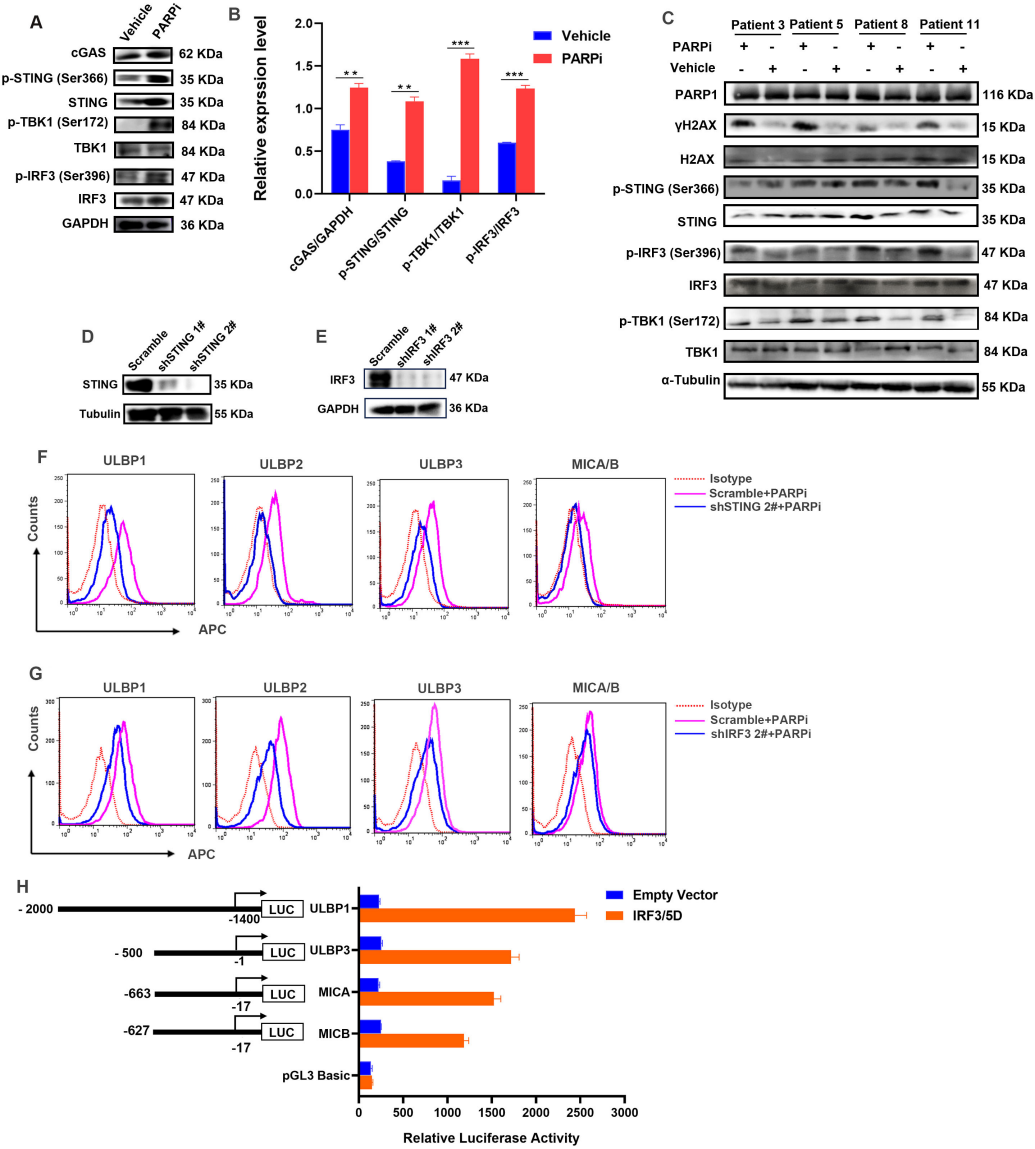
The cGAS-STING signaling is involved in the regulation of NKG2D ligands in LSCs. **(A)** WB was used to detect the protein level of cGAS, STING, phosphorylated-STING, TBK1, phosphorylated-TBK1, IRF3, and phosphorylated-IRF3 in LSCs pretreated with 1 μM PARPi or Vehicle for 6 hours. GAPDH was employed as an internal loading control. **(B)** Quantification of the WB shown in **(A)**. The Integrated Optical Density of bands was measured by the software ImageJ. Data was presented as mean ± SD from three experiments. **(C)** Primary LSCs isolated from patient 3, 5, 8 and 11 were treated with 1 μM PARPi as **(A)**, and then the indicated protein levels were detected by WB. α-Tubulin was employed as an internal loading control. **(D)** WB was used to detect the knockdown effect of STING and IRF3 **(E)** in LSCs. α-Tubulin and GAPDH were employed as internal loading control, respectively. **(F)** The expression level of NKG2D ligands on the cell surface of STING-knockdown or IRF3-knockdown **(G)** LSCs pretreated with 1 μM PARPi for 6 hours were detected by flow cytometry (BD FACSCalibur). Scramble was used as shRNA control. Mouse IgG-APC was used as isotype control. **(H)** HEK293T cells were co-transfected with ULBP1-, ULBP3-, MICA- or MICB-promoter reporter plasmid and IRF3/5D or empty vector for 24 hours, *Renilla* luciferase reporter plasmid pRL-TK was used as an internal control. DLR assay was performed to determine the reporter activity. Data was shown as means ± standard deviations from three independent experiments.

The accumulation of damaged DNA in the cytoplasm will activate the cytosolic DNA sensors. We therefore assessed their activation and found that the cGAS expression level and phosphorylated STING were significantly increased upon the treatment of PARP1 inhibitor in LSCs; meanwhile, the downstream major signals including TBK1 and IRF3 were also found to be activated with phosphorylated forms increased (**Figures 5A, B**; **Supplementary Figure S8**). Besides, the activation of cGAS/STING/IRF3 signaling upon the treatment of PARPi was also checked in primary LSCs isolated from AML patients. As shown in **Figure 5C**, the treatment of PARPi significantly increased γH2AX expression, as well as the phosphorylated-STING, TBK1 and IRF3 in primary LSCs (**Supplementary Figure S9**), which were consistent with the results from LSCs cell line. Taken together, it was suggested that the inhibition of PARP1 in LSCs could stimulate the activation of cGAS-STING signaling pathway.

To elucidate whether the cGAS-STING signaling pathway was involved in the upregulation of NKG2D ligands in LSCs mediated by PAPR1 inhibition, we constructed various shRNA targeting STING and IRF3, respectively (**Figures 5D, E**; **Supplementary Figures S10, S11**). Results from flow cytometry indicated that, in wild type LSCs, the treatment of PARP1 inhibitor could upregulate NKG2D ligands expression on cell surface; while in STING or IRF3 knock-down cells, the upregulation effects were not observed upon the inhibition of PARP1 (**Figures 5F, G**). IRF3 is an important transcriptional regulatory factor that is involved in type I interferon (IFN-I)-mediated innate immune response via a direct binding to the promoter of targeting gene. To determine whether IRF3 also acted as a transcription factor to regulate the transcription of NKG2D ligand genes, we predicted the binding sequences for IRF3 in their promoter regions in the JASPAR database (**Supplementary Figure S12**). According to the predicted results, we constructed luciferase reporter genes system to perform the DLR assays to measure the ULBP1, ULBP3, MICA and MICB promoter activity stimulated by IRF3/D5 (an activated form of IRF3). As shown in **Figure 5H**, transfection of IRF3/5D plasmid could significantly stimulate the activation of ULBP1, ULBP3, MICA and MICB promoter, indicating that IRF3 is a potential transcriptional regulatory factor for the transcription of NKG2D ligands. All these data demonstrated that the inhibition of PARP1 could activate the cGAS/STING/IRF3 signaling pathway and subsequently promote the expression of NKG2D ligands in LSCs.

## 4 Discussion

As one of the most important immune effectors in system, NK cells exert their cytolytic activities through a delicate balance between the inhibitory and activating receptors on cell surface (29). The NKG2D receptor-ligands axis is a potential target for exploring new therapeutic strategies. The induction and upregulation of NKG2D ligands on tumor cell surface allows for the targeted recognition and elimination by NK cells through the binding to NKG2D receptor. However, during tumor progression, these cells always develop strategies to downregulate NKG2D ligands expression on cell surface and avoid immune recognition by NK cells. AML, especially LSCs, the lack of NKG2D ligands on cell surface is involved in protecting leukemia cells from NK-mediated eradication, resulting in the promotion of leukemia cells infiltrating in bone marrow, liver, and spleen (30). The existence of LSCs is supposed to be facilitated for the disease relapse in AML patients after achieving remission (24), therefore, the targeting of LSCs is demonstrated to be an efficient therapeutic strategy against AML patients.

The discovery of the underlying regulatory mechanisms involved in NKG2D ligands expression in LSCs is critical for the development of NK cell-based immunotherapy. Epigenetic modification like methylation is widely implicated in the regulatory of NKG2D ligands expression in AML (10, 31). Besides, acetylation is also suggested to be associated with the modulation of NKG2D ligands in AML, since some HDAC inhibitors were reported to increase the expression of these ligands (32). Proto-oncogene c-Myc (33) and transcription factor krüppel-like factor 4 (KLF4) (34) are demonstrated to enhance the transcription of ULBP1/2/3 and MICA, respectively in AML by binding to their promoters. Moreover, the activation of nuclear factor-kappa-B (NF-κB) (35) and inhibition of signal transducer and activator of transcription 3 (STAT3) (36, 37) are also found to increase NKG2D ligands expression in AML. However, there is limited evidence to reveal the regulatory mechanisms associated with the NKG2D ligands expression in LSCs.

PARP1 is the most abundant member in the PARP superfamily and plays an essential role in the maintenance of genomic stability. The expression of PARP1 protein is elevated in various tumor tissues, which may contribute to deterioration, metastasis, and angiogenesis in tumors. Therefore, PARP1 is predicted to be an attractive anticancer therapeutic target in cancers, as well as in AML patients (38, 39). In our study, we not only found PARP1 was highly expressed in primary AML cells from BM (**Figure 3F**), but also discovered its negative correlation with NKG2D ligands expression level in AML (**Figures 3D, E**). We suggested that the cGAS-STING signaling pathway evoked by the DNA damage response was involved in the induction of NKG2D ligands in LSCs, which was initiated by the inhibition of PARP1. cGAS is the major cytosolic DNA sensor that detects double-stranded DNA, which subsequently catalyzes the synthesis of 2′3′-cyclic GMP-AMP (cGAMP), a secondary messenger that binds to STING and activates downstream IRF3 and NF-κB signals to produce various cytokines. We supposed that when PARP1 was inhibited by either small interfering RNA or inhibitor, the DNA repair events would be interrupted, resulting in the accumulation of aberrant DNA to activate the cGAS-STING-IRF3 signaling pathway in LSCs. Under the treatment of PAPRi, we had observed the increased protein level of γH2AX (**Figures 4C, D**, **5C**), which was a sensitive molecular marker of DNA damage, but unfortunately, how these damaged DNA triggered the activation of cGAS was still unclear in our study. However, we might find some clues according to the previous studies, and two main points were summarized until now (40). On the one hand, it was considered that during the DNA damage process, genomic instability leaded to the release of small fragments of DNA from the nucleus, which would further activate cGAS in the cytoplasm (41); on the other hand, it was supposed that cGAS could transfer into the nucleus and co-localize with damaged dsDNA, leading to its activation (42). Although we had observed the activation of downstream signal in STING, more studies are required to confirm this issue in the future. IRF3 is a critical transcription factor that is implicated in the induction of interferons in the antiviral signaling pathways (43), as well as in the production of NKG2D ligands in mice lymphoma (15, 44). But its role in regulating NKG2D ligands expression in human LSCs has not been revealed. In our study, the knock-down of IRF3 abolished the influence of PARPi on the upregulation of NKG2D ligands in LSCs (**Figure 5G**). Moreover, a direct regulatory role of IRF3 was observed on the promoters of NKG2D ligand genes. The activated IRF3 (IRF3/5D) could stimulate the promoter activity of NKG2D ligand genes (**Figure 5H**), indicating that IRF3 is necessary for the induction of NKG2D ligands in LSCs through the DNA sensor pathway. The application of PARPi in cancers not only promotes the tumor cells to death (45), but also contributes to the upregulation of NKG2D ligands in LSCs, which further sensitizes LSCs to NK cell-mediated cytotoxicity.

Altogether, our study shed lights on the immune evasion strategy applied by AML to evade NK cell-mediated immune killing by downregulation NKG2D ligands expression on LSCs cell surface. The discovery of cGAS-STING-IRF3 signaling pathway associated with the regulation of NKG2D ligands in LSCs will provide novel insights for exploring NK cell-based immunotherapies against AML by developing sensitizers for LSCs to evoke the activity of NK cells.

## Data Availability

The original contributions presented in the study are included in the article/**Supplementary Material**. Further inquiries can be directed to the corresponding author.

## References

[1] Siegel RL Kratzer TB Giaquinto AN Sung H Jemal A . Cancer statistics, 2025. CA Cancer J Clin. (2025) 75:10–45. doi: 10.3322/caac.21871, PMID: 39817679 PMC11745215

[2] Sabbath KD Ball ED Larcom P Davis RB Griffin JD . Heterogeneity of clonogenic cells in acute myeloblastic leukemia. J Clin Invest. (1985) 75:746–53. doi: 10.1172/JCI111756, PMID: 3855866 PMC423572

[3] Griffin JD Lowenberg B . Clonogenic cells in acute myeloblastic leukemia. Blood. (1986) 68:1185–95. doi: 10.1182/blood.V68.6.1185.1185 3535923

[4] Wang X Huang S Chen JL . Understanding of leukemic stem cells and their clinical implications. Mol Cancer. (2017) 16:2. doi: 10.1186/s12943-016-0574-7, PMID: 28137304 PMC5282926

[5] Li SQ Xu LP Wang Y Zhang XH Chen H Chen YH . Leukemia stem cells for relapse prediction in AML patients receiving allografts: long-term follow-up of a prospective study. Bone Marrow Transplant. (2025) 60:1472–9. doi: 10.1038/s41409-025-02699-8, PMID: 40849363

[6] Sugimoto E Li J Hayashi Y Iida K Asada S Fukushima T . Hyperactive Natural Killer cells in Rag2 knockout mice inhibit the development of acute myeloid leukemia. Commun Biol. (2023) 6:1294. doi: 10.1038/s42003-023-05606-3, PMID: 38129572 PMC10739813

[7] Zhang L Zhao Y Dong Y Jiang X . NK cell-based immunotherapy strategies for myeloid leukemia. Front Immunol. (2025) 16:1621885. doi: 10.3389/fimmu.2025.1621885, PMID: 40726987 PMC12301322

[8] Restelli C Ruella M Paruzzo L Tarella C Pelicci PG Colombo E . Recent advances in immune-based therapies for acute myeloid leukemia. Blood Cancer Discov. (2024) 5:234–48. doi: 10.1158/2643-3230.BCD-23-0202, PMID: 38904305 PMC11215380

[9] Dhar P Wu JD . NKG2D and its ligands in cancer. Curr Opin Immunol. (2018) 51:55–61. doi: 10.1016/j.coi.2018.02.004, PMID: 29525346 PMC6145810

[10] Baragano Raneros A Martin-Palanco V Fernandez AF Rodriguez RM Fraga MF Lopez-Larrea C . Methylation of NKG2D ligands contributes to immune system evasion in acute myeloid leukemia. Genes Immun. (2015) 16:71–82. doi: 10.1038/gene.2014.58, PMID: 25393931

[11] Donato E Correia N Andresen C Karpova D Wurth R Klein C . Retained functional normal and preleukemic HSCs at diagnosis are associated with good prognosis in DNMT3AmutNPM1mut AMLs. Blood Adv. (2023) 7:1011–8. doi: 10.1182/bloodadvances.2022008497, PMID: 36453648 PMC10036514

[12] Sauer M Reiners KS Hansen HP Engert A Gasser S von Strandmann EP . Induction of the DNA damage response by IAP inhibition triggers natural immunity via upregulation of NKG2D ligands in Hodgkin lymphoma *in vitro*. Biol Chem. (2013) 394:1325–31. doi: 10.1515/hsz-2013-0161, PMID: 23787466

[13] Liu C Lai H Chen T . Boosting natural killer cell-based cancer immunotherapy with selenocystine/transforming growth factor-beta inhibitor-encapsulated nanoemulsion. ACS Nano. (2020) 14:11067–82. doi: 10.1021/acsnano.9b10103, PMID: 32806028

[14] Gasser S Orsulic S Brown EJ Raulet DH . The DNA damage pathway regulates innate immune system ligands of the NKG2D receptor. Nature. (2005) 436:1186–90. doi: 10.1038/nature03884, PMID: 15995699 PMC1352168

[15] Lam AR Bert NL Ho SS Shen YJ Tang LF Xiong GM . RAE1 ligands for the NKG2D receptor are regulated by STING-dependent DNA sensor pathways in lymphoma. Cancer Res. (2014) 74:2193–203. doi: 10.1158/0008-5472.CAN-13-1703, PMID: 24590060 PMC4229084

[16] Weiss T Schneider H Silginer M Steinle A Pruschy M Polic B . NKG2D-dependent antitumor effects of chemotherapy and radiotherapy against glioblastoma. Clin Cancer Res. (2018) 24:882–95. doi: 10.1158/1078-0432.CCR-17-1766, PMID: 29162646

[17] Cuce M Gallo Cantafio ME Siciliano MA Riillo C Caracciolo D Scionti F . Trabectedin triggers direct and NK-mediated cytotoxicity in multiple myeloma. J Hematol Oncol. (2019) 12:32. doi: 10.1186/s13045-019-0714-9, PMID: 30898137 PMC6429746

[18] Soriani A Zingoni A Cerboni C Iannitto ML Ricciardi MR Di Gialleonardo V . ATM-ATR-dependent up-regulation of DNAM-1 and NKG2D ligands on multiple myeloma cells by therapeutic agents results in enhanced NK-cell susceptibility and is associated with a senescent phenotype. Blood. (2009) 113:3503–11. doi: 10.1182/blood-2008-08-173914, PMID: 19098271

[19] Chappidi N Quail T Doll S Vogel LT Aleksandrov R Felekyan S . PARP1-DNA co-condensation drives DNA repair site assembly to prevent disjunction of broken DNA ends. Cell. (2024) 187:945–961.e918. doi: 10.1016/j.cell.2024.01.015, PMID: 38320550

[20] Zhu H Wang B Kong L An T Li G Zhou H . Parvifoline AA promotes susceptibility of hepatocarcinoma to natural killer cell-mediated cytolysis by targeting peroxiredoxin. Cell Chem Biol. (2019) 26:1122–1132.e1126. doi: 10.1016/j.chembiol.2019.04.003, PMID: 31130519

[21] Chang TH Liao CL Lin YL . Flavivirus induces interferon-beta gene expression through a pathway involving RIG-I-dependent IRF-3 and PI3K-dependent NF-kappaB activation. Microbes Infect. (2006) 8:157–71. doi: 10.1016/j.micinf.2005.06.014, PMID: 16182584

[22] Zhu H Zheng C Xing J Wang S Li S Lin R . Varicella-zoster virus immediate-early protein ORF61 abrogates the IRF3-mediated innate immune response through degradation of activated IRF3. J Virol. (2011) 85:11079–89. doi: 10.1128/JVI.05098-11, PMID: 21835786 PMC3194975

[23] Zhu H Wang F Ju X Kong L An T Zhao Z . Aurovertin B sensitizes colorectal cancer cells to NK cell recognition and lysis. Biochem Biophys Res Commun. (2018) 503:3057–63. doi: 10.1016/j.bbrc.2018.08.093, PMID: 30144974

[24] Stelmach P Trumpp A . Leukemic stem cells and therapy resistance in acute myeloid leukemia. Haematologica. (2023) 108:353–66. doi: 10.3324/haematol.2022.280800, PMID: 36722405 PMC9890038

[25] Vetrie D Helgason GV Copland M . The leukaemia stem cell: similarities, differences and clinical prospects in CML and AML. Nat Rev Cancer. (2020) 20:158–73. doi: 10.1038/s41568-019-0230-9, PMID: 31907378

[26] Landerer H Arnone M Wieboldt R Goersch E Stanger AMP Konantz M . Two flow cytometric approaches of NKG2D ligand surface detection to distinguish stem cells from bulk subpopulations in acute myeloid leukemia. J Vis Exp. (2021) 168. doi: 10.3791/61803-v, PMID: 33682851

[27] Zhou H Qin D Xie C Zhou J Jia S Zhou Z . Combinations of HDAC inhibitor and PPAR agonist induce ferroptosis of leukemic stem cell-like cells in acute myeloid leukemia. Clin Cancer Res. (2024) 30:5430–44. doi: 10.1158/1078-0432.CCR-24-0796, PMID: 39321217

[28] Pandey N Black BE . Rapid detection and signaling of DNA damage by PARP-1. Trends Biochem Sci. (2021) 46:744–57. doi: 10.1016/j.tibs.2021.01.014, PMID: 33674152 PMC8364484

[29] Chen S Zhu H Jounaidi Y . Comprehensive snapshots of natural killer cells functions, signaling, molecular mechanisms and clinical utilization. Signal Transduct Target Ther. (2024) 9:302. doi: 10.1038/s41392-024-02005-w, PMID: 39511139 PMC11544004

[30] She M Niu X Chen X Li J Zhou M He Y . Resistance of leukemic stem-like cells in AML cell line KG1a to natural killer cell-mediated cytotoxicity. Cancer Lett. (2012) 318:173–9. doi: 10.1016/j.canlet.2011.12.017, PMID: 22198207

[31] Liu M Du M Yu J Qian Z Gao Y Pan W . CEBPA mutants down-regulate AML cell susceptibility to NK-mediated lysis by disruption of the expression of NKG2D ligands, which can be restored by LSD1 inhibition. Oncoimmunology. (2022) 11:2016158. doi: 10.1080/2162402X.2021.2016158, PMID: 35003895 PMC8741297

[32] Diermayr S Himmelreich H Durovic B Mathys-Schneeberger A Siegler U Langenkamp U . NKG2D ligand expression in AML increases in response to HDAC inhibitor valproic acid and contributes to allorecognition by NK-cell lines with single KIR-HLA class I specificities. Blood. (2008) 111:1428–36. doi: 10.1182/blood-2007-07-101311, PMID: 17993609

[33] Nanbakhsh A Pochon C Mallavialle A Amsellem S Bourhis JH Chouaib S . c-Myc regulates expression of NKG2D ligands ULBP1/2/3 in AML and modulates their susceptibility to NK-mediated lysis. Blood. (2014) 123:3585–95. doi: 10.1182/blood-2013-11-536219, PMID: 24677544 PMC4198341

[34] Alkhayer R Ponath V Frech M Adhikary T Graumann J Neubauer A . KLF4-mediated upregulation of the NKG2D ligand MICA in acute myeloid leukemia: a novel therapeutic target identified by enChIP. Cell Commun Signal. (2023) 21:94. doi: 10.1186/s12964-023-01118-z, PMID: 37143070 PMC10157933

[35] Wu HY Li KX Pan WY Guo MQ Qiu DZ He YJ . Venetoclax enhances NK cell killing sensitivity of AML cells through the NKG2D/NKG2DL activation pathway. Int Immunopharmacol. (2022) 104:108497. doi: 10.1016/j.intimp.2021.108497, PMID: 34999394

[36] Zhu Z Bai Y Lu X Ding J Qi C . Rapamycin downregulates NKG2D ligands in acute myeloid leukemia cells via an activation of the STAT3 pathway: a potential mechanism for rapamycin-induced immune escape in leukemia. Transl Cancer Res. (2019) 8:473–82. doi: 10.21037/tcr.2019.03.01, PMID: 35116779 PMC8798175

[37] Zhu Z Lu X Jiang L Sun X Zhou H Jia Z . STAT3 signaling pathway is involved in decitabine induced biological phenotype regulation of acute myeloid leukemia cells. Am J Transl Res. (2015) 7:1896–907., PMID: 26692933 PMC4656766

[38] Padella A Ghelli Luserna Di Rora A Marconi G Ghetti M Martinelli G Simonetti G . Targeting PARP proteins in acute leukemia: DNA damage response inhibition and therapeutic strategies. J Hematol Oncol. (2022) 15:10. doi: 10.1186/s13045-022-01228-0, PMID: 35065680 PMC8783444

[39] Kontandreopoulou CN Diamantopoulos PT Tiblalexi D Giannakopoulou N Viniou NA . PARP1 as a therapeutic target in acute myeloid leukemia and myelodysplastic syndrome. Blood Adv. (2021) 5:4794–805. doi: 10.1182/bloodadvances.2021004638, PMID: 34529761 PMC8759124

[40] Shen R Liu D Wang X Guo Z Sun H Song Y . DNA damage and activation of cGAS/STING pathway induce tumor microenvironment remodeling. Front Cell Dev Biol. (2021) 9:828657. doi: 10.3389/fcell.2021.828657, PMID: 35265630 PMC8900217

[41] Hintzsche H Hemmann U Poth A Utesch D Lott J Stopper H . Fate of micronuclei and micronucleated cells. Mutat Res Rev Mutat Res. (2017) 771:85–98. doi: 10.1016/j.mrrev.2017.02.002, PMID: 28342454

[42] Liu H Zhang H Wu X Ma D Wu J Wang L . Nuclear cGAS suppresses DNA repair and promotes tumorigenesis. Nature. (2018) 563:131–6. doi: 10.1038/s41586-018-0629-6, PMID: 30356214

[43] Al Hamrashdi M Brady G . Regulation of IRF3 activation in human antiviral signaling pathways. Biochem Pharmacol. (2022) 200:115026. doi: 10.1016/j.bcp.2022.115026, PMID: 35367198

[44] Le Bert N Lam AR Ho SS Shen YJ Liu MM Gasser S . STING-dependent cytosolic DNA sensor pathways regulate NKG2D ligand expression. Oncoimmunology. (2014) 3:e29259. doi: 10.4161/onci.29259, PMID: 25114832 PMC4126837

[45] Baer MR Kogan AA Bentzen SM Mi T Lapidus RG Duong VH . Phase I clinical trial of DNA methyltransferase inhibitor decitabine and PARP inhibitor talazoparib combination therapy in relapsed/refractory acute myeloid leukemia. Clin Cancer Res. (2022) 28:1313–22. doi: 10.1158/1078-0432.CCR-21-3729, PMID: 35091444 PMC8976746

