# Supplementary material for: cGAS/STING-mediated upregulation of NKG2D ligands in LSCs contributes to enhanced sensitivity to NK cells
Source: Front Oncol. 2026 Feb 23;16:1764427. doi: 10.3389/fonc.2026.1764427 (PMC12967932; doi:10.3389/fonc.2026.1764427)
Supplement: Supplementary file 1 [file DataSheet1.pdf]

Supplementary Information

Table of contents

I. Supplementary materials and methods

Materials and reagents.

Clinical information for AML patients.

Flow cytometry analysis of LSCs and NK cells.

Prediction of IRF3 binding sequences.

II. Supplementary results

Supplementary Table 1. Clinical Patient information.

Supplementary Figure 1. The expression level of CD34 and CD38 in KG-1α cells before and after MACS sorting.

Supplementary Figure 2. PARPi upregulates the transcription of NKG2D ligands in LSCs.

Supplementary Figure 3. The expression level of CD34 and CD38 in primary LSCs after MACS sorting.

Supplementary Figure 4. The expression level of CD56 and CD3 in PBMC before and after MACS sorting.

Supplementary Figure 5. The Raw WB data for Figure 3F.

Supplementary Figure 6. The Raw WB data for Figure 4A.

Supplementary Figure 7. The Raw WB data for Figure 4D.

Supplementary Figure 8. The Raw WB data for Figure 5A.

Supplementary Figure 9. The Raw WB data for Figure 5C.

Supplementary Figure 10. The Raw WB data for Figure 5D.

Supplementary Figure 11. The Raw WB data for Figure 5E.

Supplementary Figure 12. The promoter information for NKG2D ligand genes.

I. Supplementary materials and methods

Materials and Reagents

Inhibitors for PARP1/2 were purchased from MCE (Olaparib, Cat#: HY-10162; Niraparib, Cat#: HY-10619). PE-CD34 monoclonal antibody (Cat#: 12-0349-42, Invitrogen), APC-CD38 monoclonal antibody (HIT2) (Cat#: 17-0389-42, Invitrogen), Labeling Check Reagents (Cat#: 130-124-695, Miltenyi Biotec), FITC-CD123 monoclonal antibody (4H11) (Cat#: 11-1239-42, Invitrogen), Human NCAM-1/CD56 APC-conjugated Antibody (Cat#: FAB2408A, R&D Systems), Human CD3 epsilon PE-conjugated Antibody (Cat#: FAB100P, R&D Systems) were used for flow cytometry analysis.

Clinical information for AML patients

The basic information including age, gender, and FAB Classification for AML patients was collected from the Electronic Medical Record System in the First Affiliated Hospital of Gannan Medical University.

Flow cytometry analysis

The purity of $CD34^{+}CD38^{-}$ LSCs derived from KG-1α cells or primary AML after sorting by MACS Separation Kit were analyzed by flow cytometry (FACS Calibur flow cytometer, BD Biosciences) through fluorescent staining with CD34-PE and CD38-APC or Labeling Check Reagents; the purity of human primary NK cells was analyzed by flow cytometry through fluorescent staining with CD56-APC and CD3-PE antibodies. Dead cells were excluded from the analysis based on scatter signals and propidium iodide fluorescence.

Prediction of IRF3 binding sequences

The promoter sequences (approximate 2, 000 bp length in the 5' upstream region of indicated gene sequence) for ULBP1, ULBP3, MICA and MICB were predicated and obtained from National Center for Biotechnology Information (NCBI). Then the IRF3 binding sites in the supposed "promoter" sequences were predicted in the JASPAR database (https://jaspar.elixir.no/). Afterward, the predicted binding sequences were marked and included in the promoter sequences which would been cloned into the pGL3-Basic vector.

II. Supplementary results

Supplementary Table 1. The basic information for AML patients.

Patient ID	Age (Years)	Gender	FAB Classification	PARP1 transcriptional level
No.1	13	Male	AML-M3	High
No.2	5	Male	AML-M3	High
No.3	10	Male	AML-M2b	Medium
No.4	13	Male	AML-M3	High
No.5	13	Male	AML-M2	High
No.6	5	Male	AML-M3	Low
No.7	8	Female	AML-M2	High
No.8	9	Female	AML-M2	High
No.9	7	Male	AML-M4	Medium
No.10	6	Male	AML-M3	Medium
No.11	10	Male	AML-M2	High
No.12	11	Male	AML-M3	Yes
No.13	12	Female	AML-M3	Medium
No.14	8	Female	AML-M3	Low
No.15	9	Male	AML-M3	High
No.16	7	Male	AML-M3	High
No.17	8	Male	AML-M2	Medium
No.18	12	Male	AML-M3	High
No.19	10	Female	AML-M3	Low

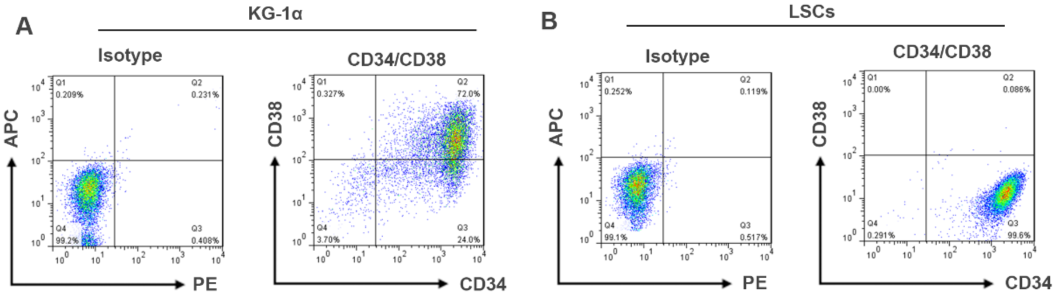

Supplementary Figure 1. The expression level of CD34 and CD38 in KG-1 α cells before and after MACS sorting.

(A) The KG-1α cells were stained with CD34-PE and CD38-APC antibodies, and then analyzed by flow cytometry. Approximate 24% KG-1α cells positively expressed CD34 along with negatively expressed CD38. (B) After MACS Separation, the cells were analyzed as (A), and the $CD34^{+}$ $CD38^{-}$ cell population accounted for 99.6%.

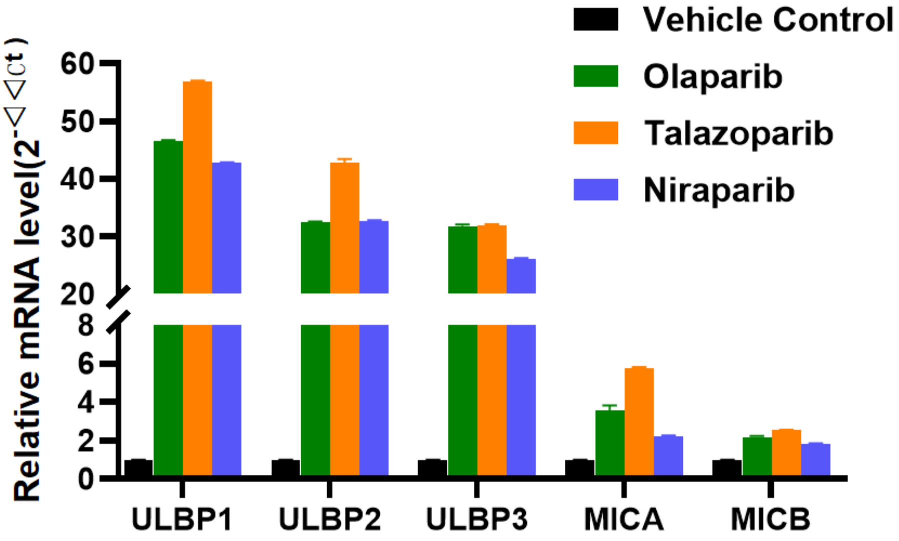

Supplementary Figure 2. PARPi upregulates the transcription of NKG2D ligands in LSCs.

LSCs were treated with the indicated PARPi at a concentration of 1 μM for 24 hours, and then cells were collected to extract total RNA. The mRNA transcriptional level of ULBP1, ULBP2, ULBP3, MICA and MICB were detected by quantitative real-time RT-PCR. Results were representative of three different experiments.

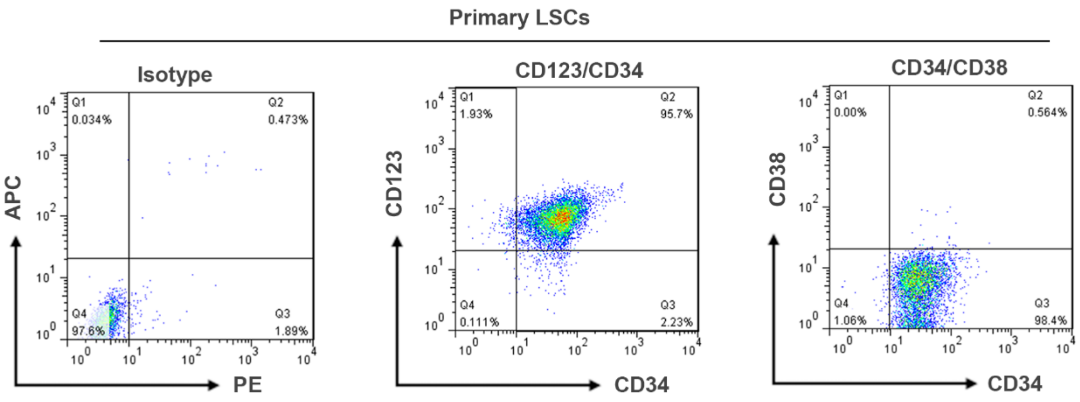

Supplementary Figure 3. The expression level of CD34 and CD38 in primary LSCs after MACS sorting.

The primary LSCs were separated from primary AML cells (patient 3, patient 5, patient 8, patient 11) via MACS Separation, and then the LSCs from these patients were mixed together to detect the expression level of CD34 and CD38 by flow cytometry through staining with CD34-PE and CD123-APC, or CD34-PE and CD38-APC antibodies.

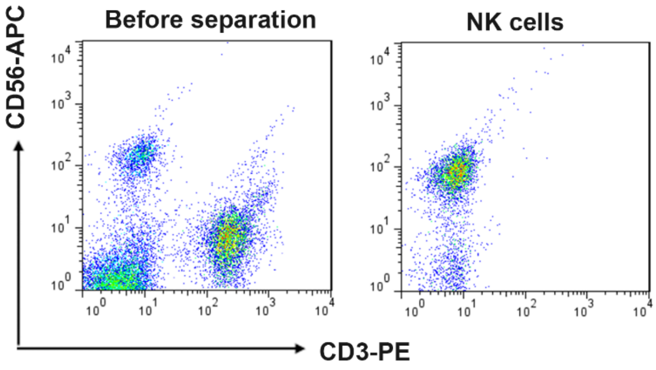

Supplementary Figure 4. The expression level of CD56 and CD3 in PBMC before and after MACS sorting.

The PBMC cells (left panel) and primary NK cells isolated from PBMC via MASC Separation (right panel) were fluorescently stained with CD56-PE and CD3-FITC and analyzed by flow cytometry.

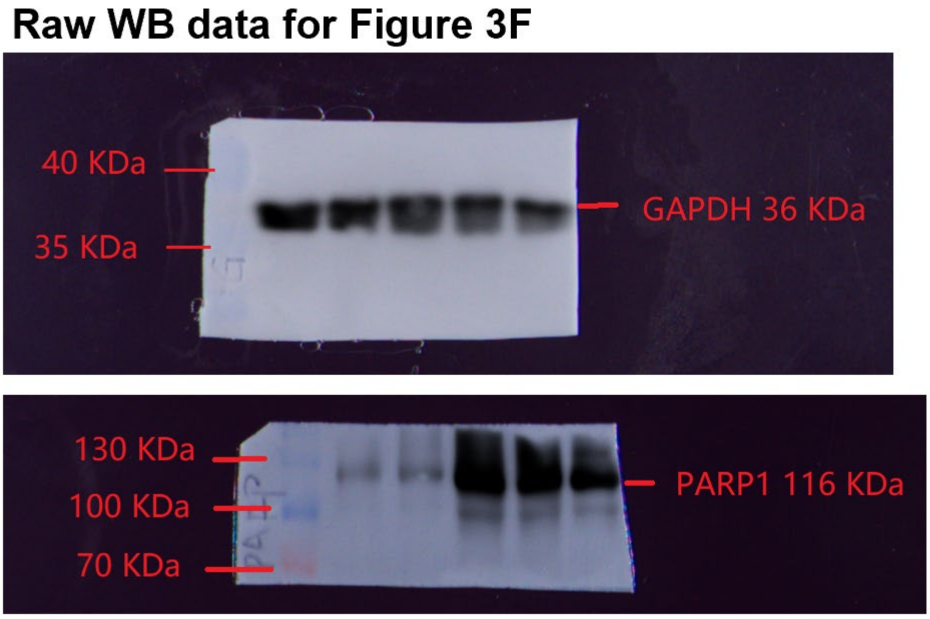

Supplementary Figure 5. The Raw WB data for Figure 3F

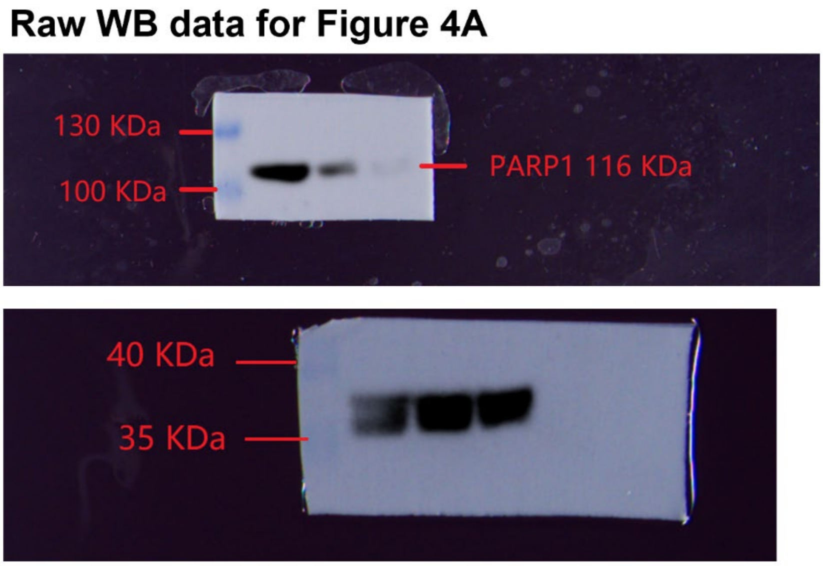

Supplementary Figure 6. The Raw WB data for Figure 4A

Raw WB data for Figure 4D

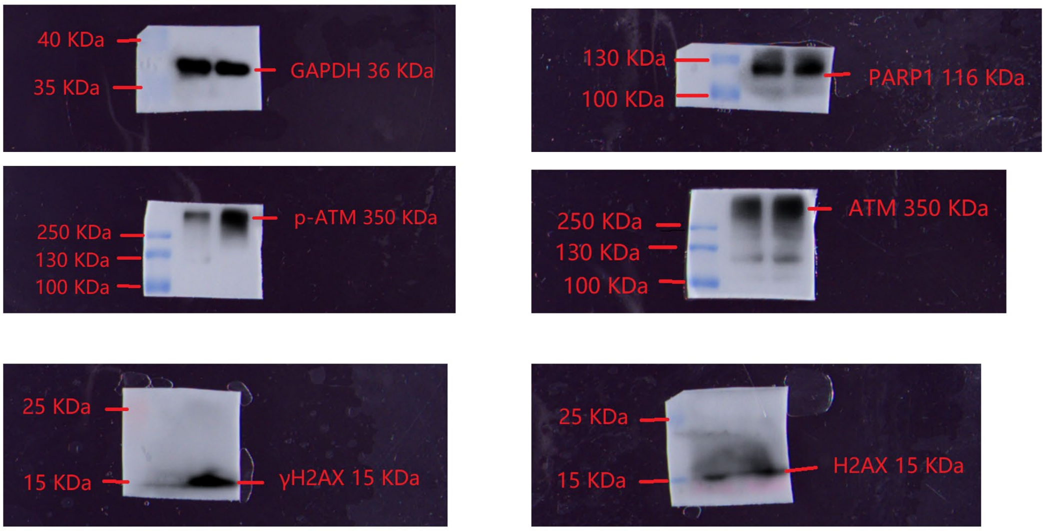

Supplementary Figure 7. The Raw WB data for Figure 4D

Raw WB data for Figure 5A

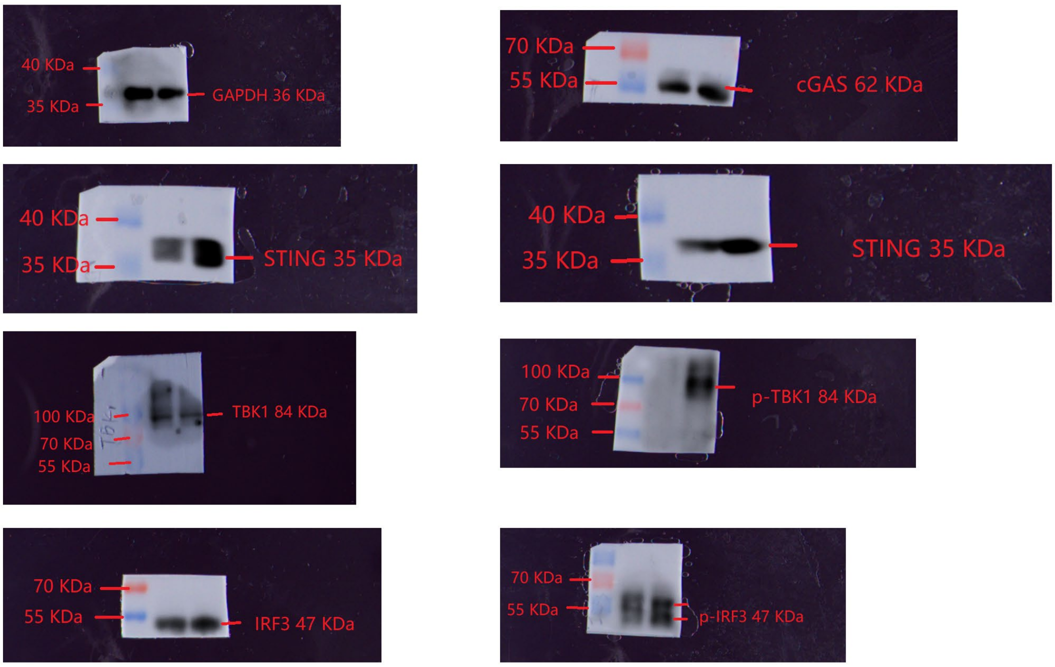

Supplementary Figure 8. The Raw WB data for Figure 5A

Raw WB data for Figure 5C

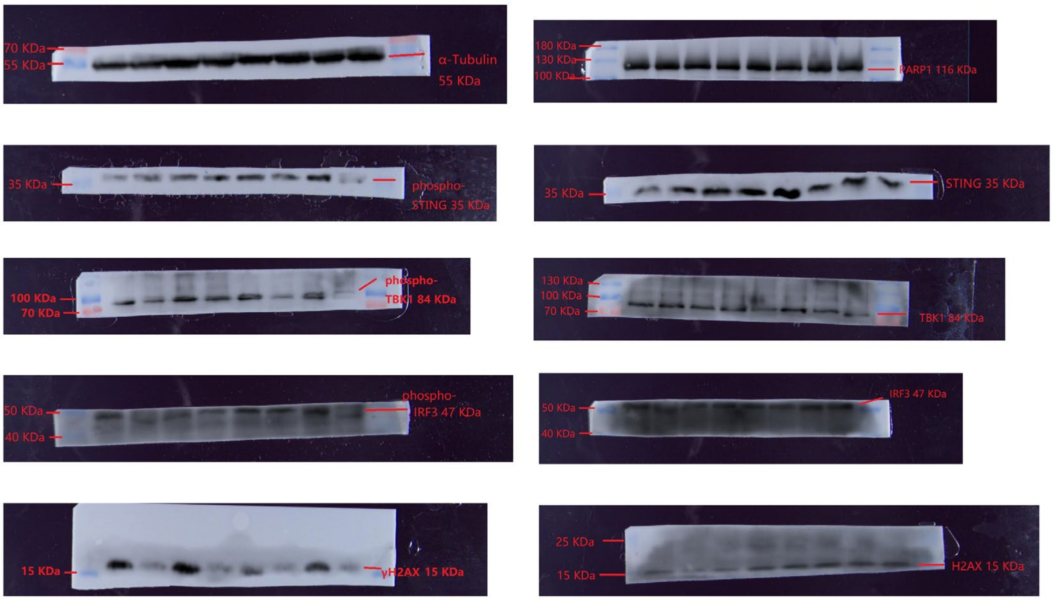

Supplementary Figure 9. The Raw WB data for Figure 5C

Raw WB data for Figure 5D

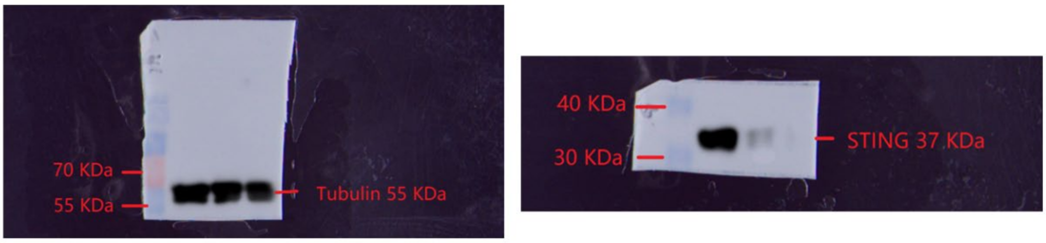

Supplementary Figure 10. The Raw WB data for Figure 5D

Raw WB data for Figure 5E

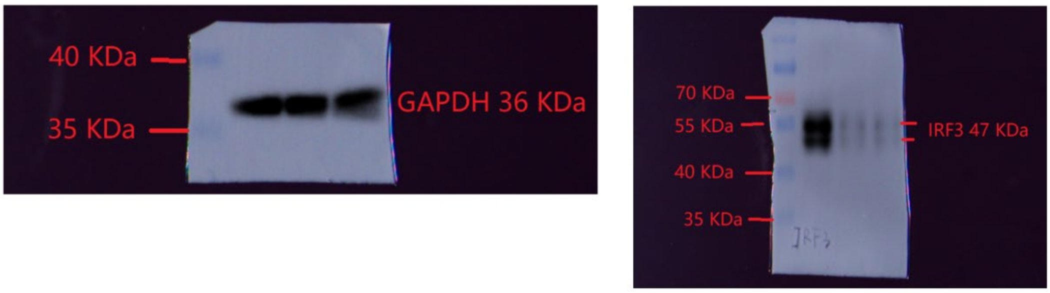

Supplementary Figure 11. The Raw WB data for Figure 5E

A

Chromosome6-Nc 000006.12

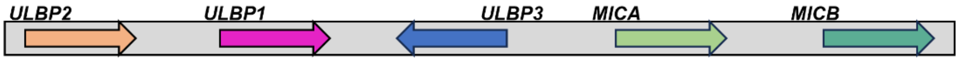

B

ULBP1 promoter

-2000 CCATTCACTCCCACAACTGTCTCATTCATTCACTGGTTCACTCAGCCTCCAACCACCCTT
AAATCATGTCAGGCTCACCAGCTGACGGAGAGAAAAATCATCCTTTGTAGGTCTGAAAA
ACGTGTCTGAATCCTGATGGTGGGTCTTGTTTTCATTTCCACCCCCTCACAGCACACTCA
GGGCCATGAAGAGGCCATGGCCTGGGCTCAGAGCTCCAGCTCTCCTCAGGGTGGGCC

ULBP3 promoter

-1 AAAACATGCCTGAGTCTTGATGGTGGGTCTTGTTTTCATTTCCTCCCCATCACAGCGCC
CCCAGGGCCATGAAGAGGCCATGGCCTGGGCTCAGAGATCCAGCTCTCCTCAGGGT
GGGCCTGGCAGTTGCTGACTTCAGGGAGAGCTGGAGGCCTCACAACTGAAATAGGA
GACCTGCCCTGGGTCCTTAGCATGTGCAAGGACAGGGTTCCTTGTAGAATCTGCTCAT

MICA promoter

-573 TGCGGGTCTGCATTTTCATAACGGGCAGGTGTGAGTGCCCTGCAGCTGGAGACCAGA
AGCCTGAAGGCAGCTCGGCCCTCCCCAGCCCACAGCGCCGTTATTCCGTTTCTATATC
AGTAAACACATTTCATTTTCCGTAGACCAGGGCGGGGTGACGGGTGATCCCAGTCCTC
GCAGTGAATTCCGGGCAGCAAAATTCAAAAC

MICB promoter

-576 GGTCTGCATTTTCACAGCGGCAGGTGTGAGTGCCGCGCAGCTGGAGACCAGAAGCCT
GAGGCAGCTCGGCCCTCCCCAGCCCAAAGTGCCGTTATTCCGTTTCTGTATCAGTAAA
CACGTTTCATTTTCCGTAGACCAGGGAAGGGTGATGGGTGATCCCAGTCCTCGCAGTG

Supplementary Figure 12. The promoter information for NKG2D ligand genes

(A) ULBP1, ULBP2, ULBP3, MICA and MICB genes were located in Chromosome6-Nc 000006-12 as shown. (B) The predicated IRF3 binding sites from JASPAR database in ULBP1, ULBP3, MICA and MICB were marked in red.
